# SARS-CoV-2 infection: a possible trigger for the recurrence of IgA nephropathy after kidney transplantation?

**DOI:** 10.1007/s40620-023-01684-y

**Published:** 2023-06-21

**Authors:** Eric Jankowski, Mandy Schlosser, Thorsten Wiech, Gunter Wolf, Martin Busch

**Affiliations:** 1 Department of Internal Medicine III, University Hospital Jena, Friedrich Schiller University, Am Klinikum 1, 07747 Jena, Germany; 2 Center for Diagnostics, Institute of Pathology, Section of Molecular Pathology and Cytopathology, University Medical Center Hamburg Eppendorf, Hamburg, Germany

## Abstract

Immunoglobulin A nephropathy, the most common primary glomerulonephritis worldwide, is a leading cause of chronic kidney disease and end-stage kidney failure. Several cases of immunoglobulin A nephropathy relapse in native kidneys have been described after COVID-19 vaccination or SARS-CoV-2 infection. Here, we report the case of a 52-year-old kidney transplant recipient who had a stable transplant function for more than 14 years, with a glomerular filtration rate above 30 ml/min/1.73 m^2^. The patient had been vaccinated against COVID-19 four times with the Pfizer-BioNTech vaccine, most recently in March 2022. Eight weeks after a symptomatic SARS-CoV-2 infection in June 2022, his glomerular filtration rate had decreased by more than 50%, and his proteinuria increased to 17.5 g per day. A renal biopsy indicated highly active immunoglobulin A nephritis. Despite steroid therapy, the function of the transplanted kidney deteriorated, and long-term dialysis became necessary because of recurrence of his underlying renal disease. This case report provides what is, to our knowledge, the first description of recurrent immunoglobulin A nephropathy in a kidney transplant recipient after SARS-CoV-2 infection leading to severe transplant failure and finally graft loss.

**Supplementary Information:**

The online version contains supplementary material available at 10.1007/s40620-023-01684-y.

## Introduction

Immunoglobulin A nephropathy (IgAN), the most common primary glomerulonephritis worldwide, remains a leading cause of chronic kidney disease and end-stage kidney disease [1, 2]. Although the effects of IgAN recurrence after kidney transplantation have been controversial in the past [3–5], recurrence has occurred in transplanted kidneys. Recurrence rates have been reported to vary between 9 and 61%, depending on the biopsy strategy and follow-up design [6].

Growing evidence indicates a link between SARS-CoV-2 infection and kidney injury in native and transplanted kidneys, thus leading to an increase in mortality [7]. Further data are needed to identify COVID-19 as a possible trigger of IgAN manifestations or increased disease activity; however, a growing number of case reports suggest a possible connection between COVID-19 and IgAN in native kidneys [8–10].

## Case report

A 52-year-old White male patient was first diagnosed with IgAN in 2007. His biopsy results at that time confirmed mesangioproliferative glomerulonephritis of IgA type with both focal-segmental and focal-global glomerulosclerosis, and 50% interstitial fibrosis/tubular atrophy. Because of progressive loss of kidney function, steroid treatment was initiated; however, kidney replacement therapy through haemodialysis became necessary in 2008. A kidney transplantation was performed in 2011 with an HLA-mismatch of 1/2/1; both the donor and recipient had negative cytomegalovirus status. The patient’s transplant function was stable, with an estimated glomerular filtration rate (eGFR) above 30 ml/min/1.73 m^2^ for more than a decade. Resection of an atypical carcinoid tumour in the left lower lobe of the lung in 2018 had no major effects on transplant function. Thereafter, the patient continued immunosuppressive therapy with mycophenolate mofetil (MMF, 2 × 500 mg per day) and tacrolimus (trough levels 5–7 ng/ml).

**Fig. 1:**
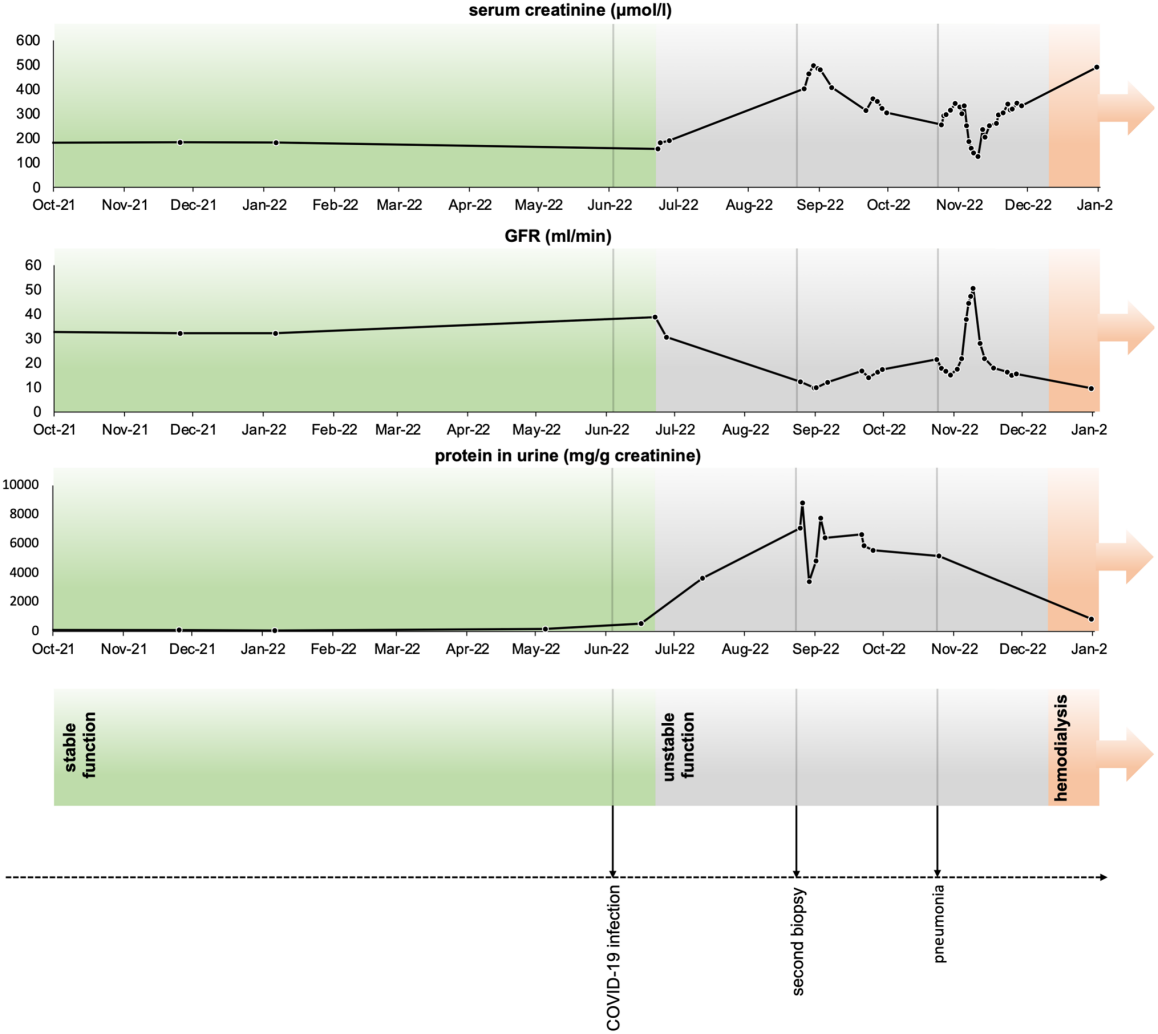
Timeline of kidney function and proteinuria, with important events highlighted

The patient was vaccinated four times against COVID-19 with the Pfizer-BioNTech vaccine, most recently in March 2022. At that time, his eGFR was stable, at 32 ml/min/1.73 m^2^, and his proteinuria was 400 mg per day at maximum (Fig. 1). Erythrocyturia was never ascertained.

On 22 June, 2022, the patient was hospitalised because of moderate SARS-CoV-2 infection with physical weakness. At that time, his urine test findings were unremarkable, and no erythrocyturia or proteinuria were observed. CT scan indicated no pulmonary involvement. A polymerase chain reaction test revealed a threshold cycle value of 13.99, and the patient’s SARS-CoV-2 IgG antibody (spike) level was 20,900 BAU/ml. His tacrolimus level was decreased from 5–7 to 3–5 ng/ml. Mycophenolate mofetil was discontinued for only 3 days and was subsequently resumed. Treatment with the monoclonal antibody combination of tixagevimab with cilgavimab (150 mg each), as well as therapy with the antiviral agent remdesivir for 3 days (200 mg intravenously at day 1; 100 mg at days 2 and 3) was initiated, and the patient’s condition improved. Dexamethasone was initially planned but was ultimately not prescribed because the patient did not require oxygen administration. On day 7, he was discharged from the hospital. At that time, an eGFR of 33 ml/min/1.73 m^2^ indicated stable transplant function, and no haematuria was detectable (Fig. 1). Immunosuppressive maintenance therapy with the previous medication (described above) was resumed without changes.

**Fig. 2:**
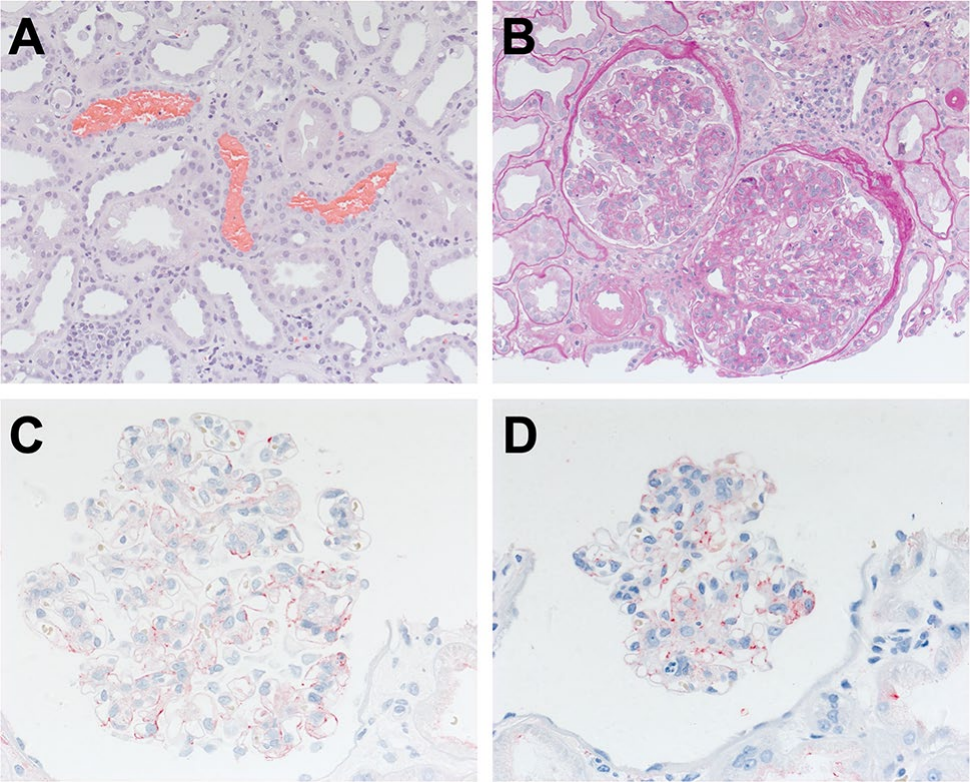
**A** H&E staining (200 ×), showing red blood cell casts in some tubular lumens. **B** Light microscopy (PAS 200 ×), revealing a mesangioproliferative pattern of glomerulonephritis with massive mesangial and endocapillary hypercellularity. **C** Immunohistochemical positivity for IgA (red) in mesangial areas and at few peripheral glomerular basement membranes (400 ×). **D** Immunohistochemical positivity for C3 (red) in mesangial areas (400 ×)

Eight weeks later, on 25 August, 2022, he was readmitted to the hospital. At that time, he had an eGFR of 12 ml/min/1.73 m^2^, proteinuria of 17.5 g per day (protein/creatinine ratio 13,430 mg/g creatinine) and haematuria of 366 erythrocytes/μl urine (3 + on a urine test strip). The patient showed volume overload and complained of progressive fatigue. A biopsy of the kidney graft indicated highly active IgA nephritis with diffuse, potentially reversible acute tubular damage, and no evidence of calcineurin inhibitor-induced tubular toxicity, or vascular or tubulointerstitial signs of rejection. C4d on peritubular capillary endothelial cells was negative. Chronic tubulointerstitial damage exceeding 50% was observed (Fig. 2). Electron microscopy revealed marked mesangial matrix increase and cell proliferation, and multiple granulocytes and monocytes adherent to the endothelium, occluding the lumen of glomerular capillaries. Most podocytes displayed foot process effacement. The peritubular capillaries showed an activated endothelium without mononuclear cell adherence. No virus particles were found.

A test for circulating HLA I and HLA II antibodies was negative. Because of his acute renal failure associated with massive proteinuria (as well as suspected transplant rejection), methylprednisolone pulses were started at a dose of 250 mg per day for 5 days, followed by oral prednisolone 30 mg per day every other day, according to a modified and dose-reduced POZZI protocol [11]. Depending on the initial response, methylprednisolone pulses were planned at months 3 and 5.

Proteinuria and albuminuria improved to a protein/creatinine ratio of 6932 mg/g creatinine and albumin/creatinine ratio of 5500 mg/g creatinine. His kidney function stabilised at an eGFR of 12 ml/min/1.73 m^2^, and he was then discharged. In October 2022, the patient was hospitalised again (Fig. 1) because of volume overload with respiratory failure and required intermittent non-invasive ventilation therapy. An attempt to establish a negative fluid balance failed and continuous haemodialysis was needed for 3 days. Pneumonia due to *Staphylococcus haemolyticus* and *Hafnia alvei* required antibiotic therapy. The patient was weaned from non-invasive ventilation, and dialysis was successfully discontinued.

Given the impaired transplant function (serum creatinine 333 µmol/l, eGFR 16 ml/min/1.73 m^2^) and the high likelihood that dialysis would be necessary in the near future, a cubital-basilica fistula was established. After being re-hospitalised 1 month later because of transplant failure and volume overload, the patient started chronic thrice-weekly haemodialysis (Fig. 1).

## Discussion

IgA nephropathy is characterised by the presence of IgA-dominant immune deposits within the glomeruli. Histological findings range from nearly normal to severe glomerulonephritis or a presentation similar to the morphology of primary focal segmental glomerulosclerosis [11]. The histological diversity is reflected by a wide range of clinical presentations: some patients undergo severe and rapidly progressive glomerulonephritis or nephrotic syndrome, whereas others experience asymptomatic micro- or macroscopic haematuria [12]. Episodic macroscopic haematuria is a hallmark clinical feature of IgAN that often spontaneously resolves within days [13].

At the time of the renal biopsy, outcomes of dialysis or death are associated with proteinuria exceeding 1 g/day, arterial hypertension and severe renal involvement, characterised by the Oxford Classification of IgAN [1, 14–16].

The pathogenesis of IgAN is not yet fully understood, but a multi-hit hypothesis has been proposed [17], ultimately resulting in complement activation [18]. Among other mechanisms, respiratory and gastrointestinal infections are associated with active IgAN [19].

SARS-CoV-2 enters the body through the respiratory tract and conjunctiva; mucosal IgA protects these barriers [20]. An IgA response to SARS-CoV-2 is detected in 75% of all patients within the first week after infection. The IgA response peaks at week 3 and appears more persistent than the IgM response [20–22]. Antigens recognised by the immune system may stimulate a cytokine storm, thereby leading to immune dysregulation and potentially triggering vasculitis; these findings support the possibility that COVID-19 might trigger IgAN [8, 19]. Vaccination against COVID-19 has been found to decrease the incidence and severity of COVID-19 among patients with kidney disease [23]. Given concerns that vaccination itself might trigger a relapse of the immunologic kidney disease, advising patients regarding risks of vaccination remains challenging [24]. Our patient received four COVID-19 vaccine doses. Recently, Canney et al. demonstrated a high relative risk, but a low increase in the absolute risk (3–5% for IgAN), of glomerular disease relapse after a second or third COVID-19 vaccine dose [25].

The primary goal of treatment for IgAN is to mitigate progression of kidney disease and proteinuria, with a treatment plan including maximum tolerated blockade of the renin-angiotensin-system, lowering blood pressure to < 120/70 mmHg, and optimizing lifestyle with dietary counselling, smoking cessation and weight control. Immunomodulating therapies are controversial [17] and are indicated only if proteinuria above 0.75–1 g/d persists after at least 90 days of optimised supportive care [26]. The clinical outcomes vary from sustained clinical remission [27] to progression of glomerular injury among patients with persistent proteinuria [1, 28].

A review published in February 2022 identified 13 cases of IgAN associated with SARS-CoV-2 infection. Ten patients showed symptoms of IgAN while still being SARS-CoV-2 positive. The mean age was 23.8 years, and the age range was 1–78 years. Most patients were treated with steroids and supportive therapy. With therapy, the condition of 10 of 12 patients with reported follow-up significantly improved, whereas two patients (in the paediatric age group) died [29]. IgAN potentially triggered by COVID-19 was observed most often in older (> 64 years) White men with a latency time between SARS-CoV-2 infection and IgAN onset ranging from 3 weeks to 9 months. Fifty percent of the patients achieved full recovery of renal function under immunosuppressive treatment and hydroxychloroquine administration [8]. Compared with the first manifestation of the disease, a relapse of IgAN is associated with a lower frequency of active proliferative glomerular lesions but more severe damage [30].

The reported rate of IgAN recurrence in transplanted kidneys varies [12]. Long-term follow up studies have indicated recurrence rates of up to 29% compared with 2–14% in studies with short-term follow-up, thus suggesting that the recurrence rate increases with the number of years after kidney transplantation [31–33]. Pre-emptive transplantation and donor-specific antibody presence at the time of transplantation are considered risk factors for IgAN recurrence. Immunoglobulin A nephropathy recurrence is associated with a 3.7-fold increase in the risk of graft loss [33].

There are scant data indicating that SARS-CoV-2 infection may trigger IgAN recurrence in transplanted kidneys, leading to graft loss. However, parallels can be drawn to patients with a poor outcome in native kidneys. In accordance with cases reported by Onate et al. [8], our patient was an older White man who presented with substantial deterioration of renal function and IgAN recurrence. Although the exact interval between SARS-CoV-2 infection and the onset of IgAN flare remains unclear, we suggest a latency of 8 weeks after infection. Given the short latency time between SARS-CoV-2 infection and IgAN recurrence, the infection appeared more likely to have triggered IgAN recurrence than the last vaccine dose, administered in March 2022.

Changes in immunosuppressive therapy can also favour IgAN recurrence. Because of SARS-CoV-2 infection, a decrease in immunosuppressive therapy became necessary in June 2022. However, immunosuppressive therapy was re-established after only 3 days, thus suggesting that a relationship between the decrease in immunosuppressive medication and IgAN recurrence was unlikely.

Similarly to findings reported by Huang et al., who described the case of a 65-year-old Chinese woman with IgAN in native kidneys after COVID-19, the kidney biopsy revealed positive staining for IgA, C3, kappa and lambda light chains, but no viral particles. In the absence of viral particles, acute kidney injury is unlikely to be a direct consequence of COVID-19 [34]. The finding of mesangial IgA positivity with segmental encroachment on the peripheral glomerular basement membrane (Fig. 1C) and the absence of viral particles supports the assumption that immune dysregulation is a major factor in IgAN after SARS-CoV-2 infection.

The patient described herein had received glucocorticoid therapy for treatment of IgAN in his native kidneys several years before, then received it again to treat IgAN recurrence in his transplanted kidney. In agreement with a report by Farooq et al., we observed an improvement in kidney function under steroid treatment in our patient. However, progressive graft failure despite immunosuppressive therapy was observed during the 6-month follow-up of our patient.

## Conclusion

This case highlights the potential consequences of SARS-CoV-2 infection in transplant recipients with IgAN.

## Supplementary Information

Below is the link to the electronic supplementary material.

Supplementary file1 (PDF 168 kb)
