# Supplementary material for: SARS-CoV-2 infection: a possible trigger for the recurrence of IgA nephropathy after kidney transplantation?
Source: J Nephrol. 2023 Jun 21;36(6):1683–7. doi: 10.1007/s40620-023-01684-y (PMC10393859; doi:10.1007/s40620-023-01684-y)
Supplement: Supplementary file 1 — Supplementary file1 (PDF 168 kb) [file 40620_2023_1684_MOESM1_ESM.pdf]

Supplementary file: hematuria

Inst. für Klin. Chemie und Laboratoriumsdiagnostik · UKJ · Am Klinikum 1 · 07740 Jena

Befundbericht

Universitätsklinikum
Zentrale Notaufnahme 1
Am Klinikum 1

07747 Jena

Fach 54

Patient: [redacted]

geb.: [redacted] **.1954 (M)**
Fall-Nr.: [redacted]
Auftrag-Nr.: [redacted]
Labor Eingang: 22.06.2022 12:39

Befundausdruck: 22.06.2022 14:44

	Untersuchung	Einheit	Ref-/Therap.-Bereich	Ergebnis 41405979 22.06.2022 12:39	Vorwerte 40872235 06.01.2022 09:59	40749259 25.11.2021 12:05	39136516 09.06.2020 11:03	38448374 24.09.2019 10:44	38135692 04.06.2019 10:41
**urine diagnostics**	**Spontanurindiagnostik**								
	**Urinstreifentest**								
pH	pH *(15)*	V1)	5 - 6	6,0 slma	6,0 bobe	**6,5** + disc	**6,5** + veec	5,5 noal	6,0 maeb
specific weight	Spez. Gew. *(15)*	g/ccm	1,01 - 1,025	1,010 slma	1,014 bobe	1,014 disc	1,012 veec	1,012 noal	1,011 maeb
leucocytes	Leukozyten *(15)*		negativ	negativ slma	negativ bobe	negativ disc	negativ veec	negativ noal	negativ maeb
blood	Blut *(15)*		negativ	negativ slma	negativ bobe	negativ disc	negativ veec	negativ noal	negativ maeb
nitrite	Nitrit *(15)*		negativ	negativ slma	negativ bobe	negativ disc	negativ veec	negativ noal	negativ maeb
protein	Protein *(15)*		negativ	+ + slma	+ + bobe	+ + disc	+ + veec	+ + noal	+ + maeb
glucose	Glucose *(15)*		normal	normal slma	normal bobe	normal disc	normal veec	normal noal	normal maeb
ketone bodies	Ketonk. *(15)*		negativ	negativ slma	negativ bobe	negativ disc	negativ veec	negativ noal	negativ maeb
urobilinogen	Urobilinogen *(15)*		normal	normal slma	normal bobe	normal disc	normal veec	normal noal	normal maeb
bilirubin	Bilirubin *(15)*		negativ	negativ slma	negativ bobe	negativ disc	negativ veec	negativ noal	negativ maeb

	Untersuchung		Einheit	Ref-/Therap.-Bereich	Ergebnis 41593675 25.08.2022 11:20	Vorwerte 41592977 25.08.2022 10:27
**Vitamins**	**Vitamine**					
vitamin B12	Vitamin B12 *(01)*	V3)	pmol/l	197 - 771	296 slma	
folic acid	Folsäure *(01)*	V3)	µg/l	3,9 - 26,8	**2,4** - slma	
vitamin D	VitD 25 *(01)*	V3)	nmol/l	>75	**37,8** - maeb	

```
Referenzbereich: Sommer: >27,8 nmol/l Winter: >14,1 nm
Empfehlung laut US-National Kidney Foundation >75 nmol
Bitte beachten Sie, dass bei Patienten, die eine Vitam
erhalten, Ergebnisse im subtherapeutischen Bereich dur
Referenzmethode, wie z. B. das LC/MS/MS-Verfahren, bes
sollten.
```

	Untersuchung		Einheit	Ref-/Therap.-Bereich	Ergebnis	Vorwerte
**urine diagnostics**	**Spontanurindiagnostik**					
	**Urinstreifentest**					
pH	pH *(15)*	V10)		5 - 6	6,0 slma	
specific weight	Spez. Gew. *(15)*		g/ccm	1,01 - 1,025	1,014 slma	
leucocytes	Leukozyten *(15)*			negativ	negativ slma	
blood	Blut *(15)*			negativ	**+++** + slma	
nitrite	Nitrit *(15)*			negativ	negativ slma	
protein	Protein *(15)*			negativ	**++** + slma	
glucose	Glucose *(15)*			normal	**++** + slma	
ketone bodies	Ketonk. *(15)*			negativ	negativ slma	
urobilinogen	Urobilinogen *(15)*			normal	normal slma	
bilirubin	Bilirubin *(15)*			negativ	negativ slma	
**urine sediment**	**Urin-Sediment**					
erythrocytes in urine	Erythrozyten im U *(15)*	V6)	/µl	<17	**366** + slma	
leucocytes in urine	Leukozyten im U *(15)*		/µl	<28	**35** + slma	
bacteria in urine	Bakterien im U *(15)*			negativ	negativ slma	
